# Prophylaxis of acute respiratory infections via improving the immune system in late preterm newborns with *E*. *coli* strain Nissle 1917: a controlled pilot trial

**DOI:** 10.1186/s40814-018-0271-y

**Published:** 2018-04-23

**Authors:** Mykola L. Aryayev, Liudmyla I. Senkivska, Nataliya K. Bredeleva, Irina V. Talashova

**Affiliations:** 1 Odessa National Medical University, 2 Valihovsky Lane, Odessa, 65082 Ukraine; 2 Odessa Regional Children Hospital, 3 Vorobyeva Str., Odessa, 65031 Ukraine; 3 Maternity Hospital No. 5, 28 Marshala Govorova Str., Odessa, 65008 Ukraine

**Keywords:** Immunity improvement, Acute respiratory infection, *E*. *coli* Nissle, Late preterm newborn, Prophylaxis

## Abstract

**Background:**

Acute respiratory infections (ARIs), caused by the high level of immaturity of the immune system, are a major cause of morbidity in preterm newborns. The probiotic *Escherichia coli* strain Nissle 1917 (EcN) is well known for its immuno-modulatory properties and may therefore enhance the immune competence. Thus, EcN administration may provide a promising possibility to decrease the risk of ARIs in this vulnerable group of children. However, clinical data supporting or refuting this hypothesis are, to our knowledge, not available. Therefore, the aim of the presented pilot trial was to collect first data on the efficacy and safety of EcN treatment to prevent ARIs in late preterm newborns.

**Methods:**

Right after birth, 62 late preterm newborns were included into an open-labeled, controlled 4-week trial with two parallel groups and a follow-up phase until the age of 1 year. All children of the treatment group received an EcN suspension orally for 3 weeks, whereas the control group was only observed. Primary efficacy variable was the number of participants with at least one ARI during the first 28 days of life. Secondary efficacy variables were the number of ARIs and the number and duration of hospitalizations caused by ARIs during the first year of life.

**Results:**

The number of participants with at least one ARI during the first 28 days of life was significantly lower in the group treated with EcN compared to that in the control group. Although only of exploratory nature, analyses of secondary efficacy variables suggest that EcN treatment may also reduce the average number of ARIs, the average number of hospitalizations caused by ARIs, and the mean duration of such hospitalizations. There is also some evidence that early EcN treatment may have long-term benefits on newborns’ health status.

**Conclusion:**

The present pilot trial provides first evidence that EcN is able to reduce the incidence of ARIs in the neonatal period of late preterm newborns. Additionally, EcN is characterized by an excellent individual biocompatibility in the absence of adverse drug reactions. Limitations of the current trial are discussed and recommendations for future confirmatory studies are made.

**Trial registration:**

ClinicalTrials.gov identifier: NCT01540162; retrospectively registered on 16 February 2012

## Background

Acute respiratory infections (ARIs) are one of the most important causes of morbidity and mortality among newborns and infants. Due to their immature immune system, newborn infants show a high vulnerability with an average of about four ARIs during the first year of life [1]. This number may be even higher in infants that were born preterm, and some data suggest that ARIs are the main reason for the rehospitalization of these children [2]. Humoral immunity deficiency in newborns is caused by insufficient transplacental transfer of IgG and a lack of IgA and IgM in serum [3]. Insufficient activation of complement systems and low concentration of its components lead to a low opsonic activity of blood and weakness of phagocytes [3]. Immaturity of bone marrow cells may often result in neutropenia [3]. Low sensitivity of T lymphocyte receptors to IL-1 and IL-2; T helper cells immaturity and poor interaction of T and B lymphocytes, resulting in low levels of antibody production in response to antigens; and insufficient production of interferon γ by CD4^+^ T cells define a weak resistance of preterm newborns against the bacterial flora, a trend to spreading of infections and a high susceptibility to viral infections [3].

A link between the newborns’ health status, the state of its intestinal flora, and immunity, respectively, was shown by several researchers [4–7]. The intestinal bacterial colonization in preterm infants depends on a number of factors—vaginal microbiota of the mother, breastfeeding profile, the conditions in the maternity hospital, perinatal pathological factors, nosocomial flora, and the use of antibiotics and other pharmaceutical agents. The microbiocoenosis is incomplete at the end of the adaptation period of preterm infants and this, in turn, is a basic principle for bacterial and viral infections [3].

In modern neonatology, probiotics are widely used in daily practice. Probiotics consist of “live microorganisms which when administered in adequate amounts confer a health benefit on the host” [8]. Several studies indicate that administering probiotics can have a favorable effect on the prevention of infections [9–13], although a series of randomized controlled trials also suggests that effects observed in one probiotic strain cannot be extrapolated to others [14, 15].

The probiotic *Escherichia coli* strain Nissle 1917 (EcN) has no pathogenic characteristics [16], is non-invasive [17], and is capable to colonize the intestinal tract for longer periods [18–20]. Due to its wide spectrum of positive and clinically relevant characteristics and its great tolerance [21–26], EcN has been approved as a medical drug under the brand name Mutaflor^®^ Suspension (Ardeypharm GmbH, Herdecke, Germany) in various countries, including the Ukraine.

One particularly interesting and important property of EcN is its broad immunomodulatory effect on the non-specific and specific immunological system. EcN stimulates the systemic production of antibodies of mucous-associated B lymphocytes and induces the systemic production of antibodies (IgM, IgA) in adults as well as in full-term and premature children [27–30]. Interestingly, early administration of secretory IgA antibodies can protect children from infections [31]. In combination, these findings make it reasonable to assume that EcN may increase the immune competence in preterm newborn infants, thereby reducing the risk of acquiring infections. However, clinical data supporting or refuting this hypothesis are, to our knowledge, not available. Therefore, the aim of the present pilot trial was to collect first data on the efficacy and safety of EcN treatment to prevent ARIs in late preterm newborns.

## Methods

### Study design and conduct

The present trial was a monocentric, prospective, and open-labelled clinical trial with a corresponding untreated control group, conducted in Odessa, Ukraine. The trial consisted of two parts, a newborn phase (time point of inclusion until day 28 of life) and a follow-up phase (day 29 of life until the end of the first year of life). Criteria for trial entry were signed informed consent of the child’s legal guardian(s), first degree of prematurity (i.e., functionally mature and gestational age 35–36 weeks), intention of the mother to exclusively breast-feed her child during the newborn phase of the trial, and a newborn’s age of 12–24 h of life at enrollment. Infants were excluded in case of non-fulfilment of one inclusion criteria, perinatal asphyxia or any perinatal pathological disease or defect (e.g., congenital birth defect, perinatal encephalopathy, other infectious disease, respiratory distress syndrome, and TORCH infections (Toxoplasmosis; Other, i.e., syphilis, varicella-zoster, parvovirus B19; Rubella; Cytomegalovirus; and Herpes infections [32])), blood analysis results with clinically significant changes (i.e., deviations from mean values larger than ± 2 SD that are probably or possibly related to any underlying pathological condition), or intention to feed the child other pro- or prebiotics during the newborn phase of the trial. Eligible preterm newborns that were born in Odessa Maternity Hospital No. 5, Odessa, Ukraine, were included in this trial. Further examinations were carried out in health care centers of the Odessa region.

Enrolled children were either assigned to the treatment group (EcN) or the control group in an alternating manner by each participating investigator. Infants of the treatment group received an orally administered daily dosage of 1 ml Mutaflor^®^ Suspension, containing 10^8^ colony-forming units of *E*. *coli* strain Nissle 1917 per milliliter, during the first 7 days of life, and 1 ml of the same suspension three times a week from day 8 to 21. Thus, the application of Mutaflor^®^ Suspension complied with the dosage scheme of the approved medical product. Infants of the control group remained untreated and were just observed.

**Fig. 1:**
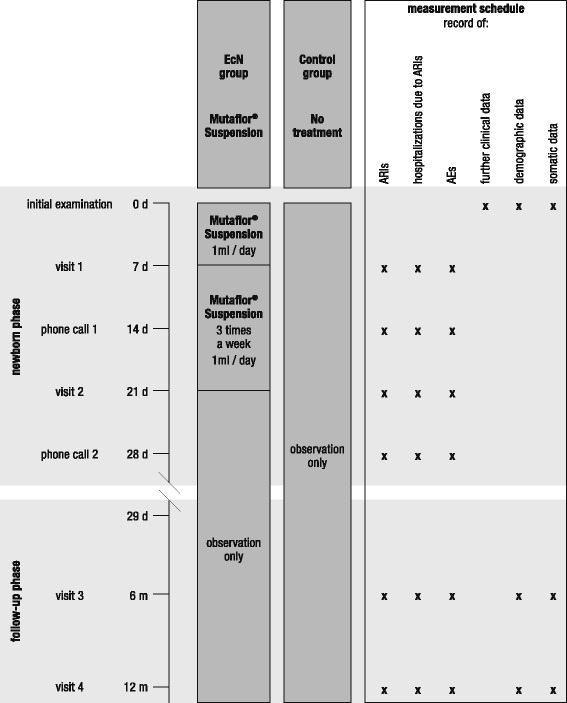
Flowchart showing the timeline of the trial and the measurement schedule. ARI acute respiratory infection

The trial comprised an initial examination (day 0), during which clinical, demographic, and somatic characteristics of the newborn were recorded (Table 1). During the newborn phase, visits were carried out on days 7 and 21, and phone contacts took place on days 14 and 28. The follow-up observations took place as part of the conventional preventive checkups carried out at the ages of 6 and 12 months in Ukraine. Efficacy and safety data were recorded based on hospital medical records (if available) and parental reporting. A flow chart is presented in Fig. 1.

**Table 1:** Baseline characteristics of children enrolled in the study of efficacy and safety of EcN for immunity improvement in late preterm newborns

Characteristics		EcN (*N* = 30)	Control (*N* = 32)
Gestational age at birth (weeks)		35.5 ± 0.5	35.5 ± 0.5
Age at inclusion (hours)		17.3 ± 3.8	17.4 ± 3.3
Sex	Female	14 (46.7)	15 (46.9)
	Male	16 (53.3)	17 (53.1)
Type of birth	Vaginal	28 (93.3)	26 (81.3)
	Caesarean	2 (6.7)	6 (18.8)
Birth weight (g)		2400 ± 122	2396 ± 77
Birth length (cm)		45.2 ± 0.8	45.2 ± 0.9
APGAR scores	I	7.5 ± 0.5	7.6 ± 0.6
	II	8.1 ± 0.3	8.1 ± 0.3

Data are mean ± SD or number (%)

*SD* standard deviation, *APGAR score* Appearance, Pulse, Grimace, Activity, Respiration score

The primary variable to assess efficacy of EcN for immunity improvement in preterm newborns was the number of participants with at least one acute respiratory infection during the newborn phase, i.e., during the first 28 days of life. Acute rhinitis, acute rhinopharyngitis, acute bronchitis, acute bronchiolitis, and pneumonia were counted as ARIs. As secondary efficacy variables, the number of ARI events and the number and duration of hospitalizations caused by ARIs during the newborn phase were documented. A further secondary objective of the present trial was to explore whether early EcN treatment has also a long-term effect on infant immunity. Therefore, all measures were also taken at the ages of 6 and 12 months.

The safety of EcN was evaluated by quantifying the number of adverse events (AEs) throughout the entire trial period, i.e., all untoward medical occurrences except the ARIs considered as primary efficacy variables. AEs were regarded as drug related if causality due to the study medication was rated “certain,” “probable,” or “possible” (according to the WHO Collaborating Centre for International Drug Monitoring) by the investigators. As a further safety measure, the somatic development, quantified by body weight and body length, was recorded at the ages of 6 and 12 months.

Figure 1 provides an overview of the points in time when efficacy and safety measures were taken.

Participants’ sufficient compliance to the trial protocol was defined as a complete lack of treatment with respect to the study medication for participants of the control group and an intake of at least 75% of the planned trial medication for participants of the treatment group.

### Statistical analysis

Counts, means, frequencies, standard deviations, and 95% confidence intervals were calculated for the recorded data according to applicability. The data used to calculate the values at 6 months of life also contained the data already recorded during the first 28 days. Similarly, reported values after 12 months of life were calculated from *all* the data collected between the time of inclusion and 12 months of life.

Categorical data were compared by applying chi-square tests with Yates’s correction and quantitative data by using the Mann-Whitney *U* tests.

The relative risk (RR), the relative risk reduction (RRR), and the number needed to treat (NNT) were calculated from the “number of participants with at least one ARI”. Analyses were performed in Statistica 7.0 or Microsoft Excel and the significance level *α* was set at *p* < 0.05.

## Results

### Participants

**Fig. 2:**
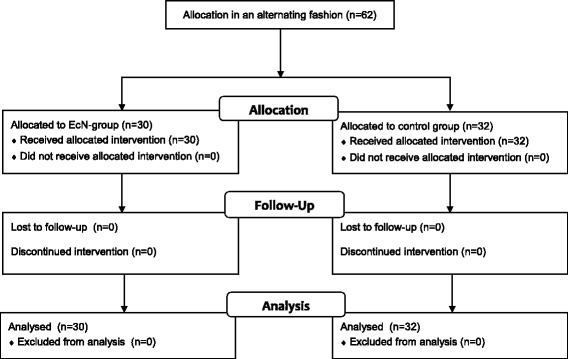
Flowchart showing the allocation of subjects

Between 19 March and 15 September 2011, a total of 62 late preterm newborns of Caucasian race were included into the trial and either assigned to the EcN (*N* = 30) or the control (*N* = 32) group (Fig. 2). The follow-up of the last participant ended on the 12 October 2012. On average, children of the two trial arms showed no substantial differences in any of the clinical, demographic, or somatic characteristics recorded during initial examination (Table 1). All participants complied with the trial protocol, such that ITT and PP analyses of all data revealed equal results.

### Efficacy and safety of EcN for immunity improvement in late preterm newborns during the first 28 days of life (newborn phase)

**Fig. 3:**
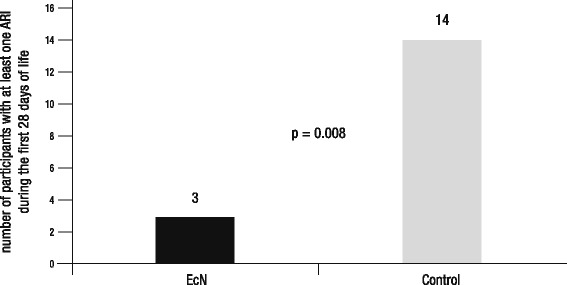
Result of primary efficacy measure. Shown are the numbers of participants with at least one ARI during the first 28 days of life for the EcN and the control group, respectively

Analysis of the primary outcome measure revealed a significant difference between the two trial arms, with fewer participants suffering from at least one ARI during the first 28 days of life in the EcN than in the control group (Fig. 3, Table 2). The risk to contract an ARI during the first month of life was 77% lower in the EcN than that in the control group, and the corresponding number needed to treat was 3.

**Table 2:** Results of the study of efficacy and safety of EcN for immunity improvement in late preterm newborns

	After 28 days of life		After 6 months of life		After 12 months of life	
	EcN (*N* = 30)	Control (*N* = 32)	EcN (*N* = 30)	Control (*N* = 32)	EcN (*N* = 30)	Control (*N* = 32)
Efficacy measures						
Total number and % of participants with at least 1 ARI	3 (10.0)	14 (43.7)	15 (50.0)	23 (71.9)	23 (76.7)	30 (93.8)
	*Χ*^2^_cor._ = 7.25; *p* = 0.008		Χ^2^_cor._ = 2.27; *p* = 0.132		Χ^2^_cor._ = 1.32; *p* = 0.251	
Mean number of ARI events	0.10 − 0.01–0.21	0.44 0.26–0.61	0.50 0.31–0.68	0.94 0.69–1.19	0.90 0.68–1.12	1.31 1.08–1.54
	*z* = 2.28, *p* = 0.02		*z* = 2.21, *p* = 0.03		*z* = 2.11, *p* = 0.03	
Mean number of hospitalizations due to ARIs	0.03 − 0.03–0.01	0.34 0.18–0.51	0.13 0.01–0.26	0.53 0.33–0.73	0.27 0.11–0.43	0.56 0.37–0.76
	*z* = 2.01, *p* = 0.04		*z* = 2.50, *p* = 0.01		*z* = 1.84, *p* = 0.07	
Mean duration of hospitalizations due to ARIs (days)	0.27 − 0.26–0.79	2.16 0.98–3.34	1.20 0.03–2.37	4.06 2.48–5.64	2.43 0.86–4.01	4.21 2.66–5.78
	*z* = 1.85, *p* = 0.06		*z* = 2.40, *p* = 0.02		*z* = 1.59, *p* = 0.11	
RR	0.23		0.70		0.82	
RRR	0.77		0.30		0.18	
NNT	3		5		6	
Safety measures						
Total number of AEs	0	6	23	21	28	30
Total number of drug- related AEs	0	0	0	0	0	0
Mean body weight (g)	N/A	N/A	6623 ± 268	6419 ± 164	9765 ± 446	9634 ± 532
Mean body length (cm)	N/A	N/A	61.4 ± 1.1	61.2 ± 0.9	73.0 ± 1.2	73.3 ± 1.4

Data are numbers (%), means and CI, means ± SD, or epidemiological indices. Means of efficacy measures were always calculated from cumulative data. For details, see the “Statistical analysis” section

*%* frequency, *CI* 95% confidence intervals, *SD* standard deviation, *ARI* acute respiratory infection, *RR* relative risk, *RRR* relative risk reduction, *NNT* number needed to treat

**Fig. 4:**
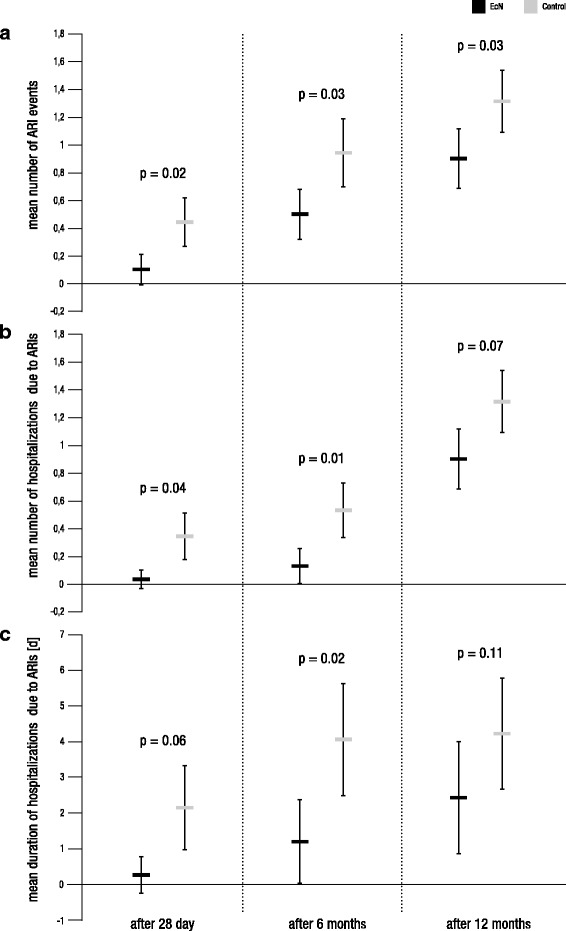
Exploratory results of secondary efficacy measures. Shown are the mean numbers of ARIs (**a**), the mean numbers of hospitalizations due to ARIs (**b**), and the mean durations of hospitalizations due to ARIs (**c**) after 28 days, 6 months, and 12 months for the EcN and the control group, respectively. Error bars represent 95% confidence intervals. As statistical analyses of secondary efficacy variables are only performed for explorative purposes, given *p* values are not corrected for α-error inflation

Furthermore, exploratory comparisons suggest that the average number of ARIs, the average number of hospitalizations caused by ARIs, and the mean duration of such hospitalizations were lower in the group treated with EcN compared to the control group (Fig. 4, Table 2). Table 3 provides an overview of the type and number of ARIs that occurred.

**Table 3:** Overview of the type and number of ARIs that occurred in the study of efficacy and safety of EcN for immunity improvement in late preterm newborns

	After 28 days of life		After 6 months of life		After 12 months of life	
	EcN	Control	EcN	Control	EcN	Control
Type of ARI						
Acute rhinitis	0	2	3	5	6	11
Acute rhinopharyngitis	2	3	7	7	11	10
Acute bronchitis	1	7	3	12	7	15
Acute bronchiolitis	0	0	1	2	1	2
Pneumonia	0	2	1	4	2	4
Total	3	14	15	30	27	42

Data are cumulative counts of occurred infections

*ARI* acute respiratory infection

During the first 28 days of life, none of the children in the EcN group suffered from an AE, whereas six adverse events were recorded in the control group (Table 2; kind of AE (no. of occurrences in EcN group/no. of occurrences in control group): infant colic (0/3); constipation (0/1); diarrhea (0/1); diaper dermatitis (0/1)).

### Efficacy and safety of EcN for immunity improvement in late preterm newborns during the first year of life (follow-up phase)

Explorative analyses of the data obtained after 6 and 12 months of life revealed that the risk to suffer from an ARI was still 30 and 18% lower in the EcN group compared to that in the untreated control group (Table 2). However, the number of participants that experienced at least one ARI did not show any meaningful difference between the EcN and the control group anymore, neither after 6 nor after 12 months of life (Table 2).

In contrast, the average number of ARI events and the average number and duration of hospitalizations caused by ARIs mostly appeared to be still different between the two trial arms, with the EcN group having a health advantage over the control group (Fig. 4, Table 2). Table 3 provides an overview of the type and number of ARIs that occurred.

The number of AEs reported during the entire trial was not considerably different (Table 2; kind of AE (no. of occurrences in EcN group/no. of occurrences in control group): *at 12 months of age*: infant colic (7/6); acute enterocolitis (2/3); constipation (5/4); diarrhea (0/1); regurgitation (2/3); acute otitis media (2/2); diaper dermatitis (0/1); atopic dermatitis (3/2); changed blood parameter (0/1); weight deficiency (2/2); iron deficiency anemia stage 1 (2/3); perinatal encephalopathy; diagnosed only 3–5 weeks after inclusion into the trial (3/2)). Importantly, the investigators judged none of the adverse events as related to treatment. Children’s somatic development, quantified by body weight and body length, was according to age [33] and comparable between the two trial arms (Table 2).

## Discussion

The present pilot trial provides first evidence that *Escherichia coli* strain Nissle 1917 is an effective and safe probiotic that may improve immune competence in late preterm newborns. The evaluation of the primary efficacy criterion revealed that treatment of late preterm newborns with EcN suspension reduced their risk to contract an ARI during the first 28 days of life by 77% in comparison to children that remained untreated, a reduction that is substantial and clinically relevant (Table 2). Thus, our findings are in line with recently published Cochrane reviews that summarize the general positive effect of probiotics in preventing the infection with acute respiratory disease [34, 35].

During the first 28 days, i.e., in a timely close correlation with the EcN administration, no adverse event could be detected in the group of children treated with EcN. Thus, EcN can be assessed as safe and well tolerated by preterm newborns with a gestational age of 35–36 weeks. In addition, the total number of adverse events reported during the first year of life was comparably low in both groups, being 28 and 30 in the EcN and the control group, respectively. None of these adverse events was judged as drug related. Similarly, children of both groups showed a normal and comparable somatic development. These data indicate that EcN treatment bears no safety risks.

EcN is able to colonize the intestinal tract. It was detected in the stool of adults up to 48 weeks [20] and in the stool of healthy newborns up to 6 months after the intake of EcN was stopped [24]. Therefore, it has been suggested that the infection prophylactic effect of EcN may even persist if the last EcN administration is already some time ago. To investigate this explorative hypothesis, variants of the primary efficacy variable, namely the numbers of children that suffered from at least one ARI during the first 6 and 12 months of life, were compared between the EcN and the control group. Interestingly, it appeared that the number of children with at least one ARI was no longer different between the EcN and the control group, neither after 6 nor after 12 months of age (Table 2). Hence, a single EcN administration period, as used in the present study, seems not sufficient to prevent children from contracting a single ARI during the first 6 months of life. Future studies may therefore consider investigating whether a constant or at least repeated administration of EcN may be able to prolong the infection-free period.

The exploratory analysis of the further secondary efficacy variables led to additional interesting observations: The average number of ARIs, the average number of hospitalizations due to ARIs, and the mean duration of these hospitalizations during the first 28 days of life all tend to be reduced by an early treatment with EcN. Overall, most of these trends seem to persist until the ages of 6 and 12 months, making it possible to speculate about a potential long-term benefit of an early postnatal EcN treatment.

Although these findings are certainly interesting and add to our knowledge of the health-promoting properties of EcN, it needs to be considered that they are just of explorative nature. Similarly, limitations in the study design, like, for example, the comparatively small sample size or the fact that the study was not randomized and open-labeled, must be taken into account when drawing conclusions from the present data. Nonetheless, we believe that our findings provide good first evidence that early-life EcN administration is able to prevent ARIs in late preterm newborns. Therefore, our study may serve as a good basis for the design of future larger trials that should be conducted randomized, blinded, and placebo-controlled in order to confirm the present findings under the highest scientific standards.

Investigators planning such studies should also consider adjustments and further development of the study protocol. For example, it would be interesting to investigate whether a constant or repeated administration of EcN during the first year of life may be able to further increase the infection prophylactic effects of EcN (see above). In addition, it might be worth to also record data on the duration and severity of the infections that occur, as these data may allow a more detailed description of the health-promoting properties of EcN. However, collection of such data may require detailed written documentation by the parents at the time of the infection (study diary) and systematic collection of medical records, in order to minimize the potential impact of recall biases. Finally, it may be recommended to also record data on other factors that are known to affect a child’s susceptibility to acquiring infections. For example, the duration of breastfeeding may act as a confounding variable in the given context and should therefore be taken into account in future studies, when analyzing the efficacy of EcN to prevent ARIs in newborns, infants, and toddlers.

## Conclusions

In summary, our results provide the first evidence that administration of EcN directly after birth can reduce the risk of contracting an ARI in late preterm newborns during the first month of their life. EcN suspension is characterized by an excellent individual tolerance and the absence of adverse drug reactions. Additional explorative findings support the impression that EcN may be an effective and safe probiotic to prevent ARIs and may provoke speculations about a potential long-term benefit of a 3-week EcN administration starting right after birth. Although the limitations of the current trial need to be considered when drawing conclusions from the present data, it may still serve as a good basis when preparing future larger trials that are required to confirm the present findings under the highest scientific standards.

## Abbreviations

ARI: Acute respiratory infection
EcN: *Escherichia coli* strain Nissle (1917)
IgA: Immunoglobulin A
IgG: Immunoglobulin G
IgM: Immunoglobulin M
IL: Interleukin

## References

[1] Kusel MM, de Klerk NH, Holt PG (2006). Role of respiratory viruses in acute upper and lower respiratory tract illness in the first year of life: a birth cohort study. Pediatr Infect Dis J.

[2] Underwood MA, Danielsen B, Gilbert WM (2007). Cost causes and rates of rehospitalization of preterm infants. J Perinatol.

[3] Volodin NN (2014). Neonatology: national guideline. short edition, GEOTAR Media.

[4] Aryayev NL, Tsiunchik YG, Varbanetz DA (2007). Clinical significance of probiotics in prophylactics and treatment of antibiotic-associated diarrhea in children. Zdorovie Rebenka.

[5] Nyankovsky SL (2010). Prebiotics and probiotics—possibilities of prophylactic and therapeutic use. Dytyachyi Likar.

[6] Maynard CL, Elson CO, Hatton RD (2012). Reciprocal interactions of the intestinal microbiota and immune system. Nature.

[7] Gao J, Wu H, Liu J (2016). Importance of gut microbiota in health and diseases of new born infants. Exp Ther Med.

[8] FAO/WHO Working Group, 2002. Guidelines for the evaluation of probiotics in food. FAO/WHO Working Group 1–11 London Ontario, Canada, Food and Agriculture Organization of the UN and World Health Organization Available at: http://www.who.int/foodsafety/fs_management/en/probiotic_guidelines.pdf. Accessed 17 Aug 2016.

[9] Hatakka K, Savilahti E, Pönkä A (2001). Effect of long term consumption of probiotic milk on infections in children attending day care centres: double blind, randomised trial. BMJ.

[10] de Vrese M, Winkler P, Rautenberg P (2005). Effect of Lactobacillus gasseri PA 16/8, Bifidobacterium longum SP 07/3, B. bifidum MF 20/5 on common cold episodes: a double blind, randomized, controlled trial. ClinNutr.

[11] Rautava S, Salminen S, Isolauri E (2009). Specific probiotics in reducing the risk of acute infections in infancy—a randomised, double-blind, placebo-controlled study. Br J Nutr.

[12] Hojsak I, Abdović S, Szajewska H (2010). Lactobacillus GG in the prevention of nosocomial gastrointestinal and respiratory tract infections. Pediatrics.

[13] Maldonado J, Cañabate F, Sempere L (2012). Human milk probiotic Lactobacillus fermentum CECT5716 reduces the incidence of gastrointestinal and upper respiratory tract infections in infants. J Pediatr Gastroenterol Nutr.

[14] Thapar N, Sanderson IR (2004). Diarrhoea in children: an interface between developing and developed countries. Lancet.

[15] Weizman Z, Asli G, Alsheikh A (2005). Effect of a probiotic infant formula on infections in child care centers: comparison of two probiotic agents. Pediatrics.

[16] Blum G, Marre R, Hacker J (1995). Properties of *Escherichia coli* strains of serotype O6. Infection.

[17] Blum-Oehler G, Oswald S, Eiteljorge K (2003). Development of strain-specific PCR reactions for the detection of the probiotic Escherichia coli strain Nissle 1917 in fecal samples. Res Microbiol.

[18] Grozdanov L, Raasch C, Schulze J (2004). Analysis of the genome structure of the nonpathogenic probiotic Escherichia coli strain Nissle 1917. J Bacteriol.

[19] Schiemann M, Sonnenborn U, Schulze J (2015). E.coli: Bedeutung in Forschung und Medizin.

[20] Joeres-Nguyen-Xuan TH, Boehm SK, Joeres L (2010). Survival of the probiotic Escherichia coli Nissle 1917 (EcN) in the gastrointestinal tract given in combination with oral mesalamine to healthy volunteers. Inflamm Bowel Dis.

[21] Sonnenborn U, Greinwald R (1991). Beziehungen zwischen Wirtsorganismus und Darmflora unter besonderer Berücksichtigung von Physiologie und Funktion der normalen Escherichia-coli-Flora.

[22] Schröder H (1992). Entwicklung der aeroben Darmflora bei Neugeborenen nach Kolonisierung mit dem E.-coli-Stamm Nissle 1917. Der Kinderarzt.

[23] Lodinová-Zádníková R, Tlaskalová-Hogenová H, Sonnenborn U (1994). Einfluss der gezielten Darmbesiedlung mit dem Escherichia-coli-Stamm Nissle 1917 auf die Immunantwort bei ausgetragenen und frühgeborenen Kindern. Der Kinderarzt.

[24] Lodinová-Zádníková R, Sonnenborn U (1997). Effect of preventive administration of a non-pathogenic Escherichia coli strain on the colonization of the intestine with microbial pathogens in newborn infants. Biol Neonate.

[25] Arribas B, Rodriguez-Cabezas ME, Camuesco D (2009). A probiotic strain of Escherichia coli, Nissle 1917, given orally exerts local and systemic anti-inflammatory effects in lipopolysaccharide-induced sepsis in mice. Br J Pharmacol.

[26] Hancock V, Dahl M, Klemm P (2010). Probiotic Escherichia coli strain Nissle 1917 outcompetes intestinal pathogens during biofilm formation. J Med Microbiol.

[27] Cukrowska B, Lodinová-Zádníková R, Enders C (2002). Specific proliferative and antibody response of premature infants to intestinal colonization with nonpathogenic probiotic E. coli strain Nissle 1917. Scand J Immunol.

[28] Lodinová-ZádníkováR, Tlaskalová-Hogenová H, Sonnenborn U (1992). Local and serum antibody response in fullterm and premature infants after artificial colonization of the intestine with. E coli strain Nissle 1917 (Mutaflor®) Pediatr Allergy Immunol.

[29] Sonnenborn U, Schulze J (2009). The non-pathogenic Escherichia coli strain Nissle 1917 features of a versatile probiotic. Microbial Ecol Health Dis.

[30] Manzhalli E, Hornuss D, Stremmel W (2016). Intestinal-borne dermatoses significantly improved by oral application of Escherichia coli Nissle 1917. World J Gastroenterol.

[31] Heikkinen T, Ruohola A, Ruuskanen O (1998). Intranasally administered immunoglobulin for the prevention of rhinitis in children. Pediatr Infect Dis J.

[32] Stegmann BJ, Carey JC (2002). TORCH Infections. Toxoplasmosis, Other (syphilis, varicella-zoster, parvovirus B19), Rubella, Cytomegalovirus (CMV), and Herpes infections. Curr Womens Health Rep.

[33] National Report on implementation of the decisions of the final outcome document of the United Nations General Assembly Special Session on children and action plan "A World Fit for Children. Ministry of Family, Youth and Sport of Ukraine State Institute for Family and Youth Development, 2007 Available at: http://www.unicef.org/arabic/worldfitforchildren/files/Ukraine_WFFC5_Report.pdf; Accessed 17 Aug 2016

[34] Bo L, Li J, Tao T, et al. Probiotics for preventing ventilator-associated pneumonia. Cochrane Database Syst Rev. 2014:CD009066. 10.1002/14651858.CD009066.pub2. 10.1002/14651858.CD009066.pub2 PMC4283465 25344083

[35] Hao Q, Dong BR, Wu T. Probiotics for preventing acute upper respiratory tract infections. Cochrane Database Syst Rev. 2015:CD006895. 10.1002/14651858.CD006895.pub2. 10.1002/14651858.CD006895.pub3 25927096

